# Supplementary material for: Mapping the evolution of fertility support policies in China: A content and instrumental analysis
Source: PLoS One. 2025 Oct 9;20(10):e0332137. doi: 10.1371/journal.pone.0332137 (PMC12510515; doi:10.1371/journal.pone.0332137)
Supplement: S1 Appendix — (ZIP) [file pone.0332137.s001.zip › S1 Appendix. 226 original policy documents/45-国家卫生健康委员会、国家发展和改革委员会关于开展全国婴幼儿照护服务示范城市创建活动的通知(附 全国婴幼儿照护服务示范城市创建活动管理办法)(FBM-CLI-4-5012681).doc]

国家卫生健康委员会、国家发展和改革委员会关于开展全国婴幼儿照护服务示范城市创建活动的通知(附：全国婴幼儿照护服务示范城市创建活动管理办法)

**发布部门：** [国家卫生健康委员会](https://www.pkulaw.com/law/chl?Aggs.IssueDepartment=60321) [国家发展和改革委员会(含原国家发展计划委员会、原国家计划委员会)](https://www.pkulaw.com/law/chl?Aggs.IssueDepartment=60301) [机构沿革](javascript:void(0))

**发文字号：**国卫人口发〔2021〕17号

**发布日期：**2021.04.30

**实施日期：**2021.04.30

**时效性：** [现行有效](https://www.pkulaw.com/law/chl?Aggs.TimelinessDic=01)

**效力级别：** [部门工作文件](https://www.pkulaw.com/law/chl?Aggs.EffectivenessDic=XE0303)

**法规类别：** [妇幼卫生](https://www.pkulaw.com/law/chl?Aggs.Category=09622) [老少妇幼残保护](https://www.pkulaw.com/law/chl?Aggs.Category=01604)

国家卫生健康委员会、国家发展和改革委员会关于开展全国婴幼儿照护服务示范城市创建活动的通知
（国卫人口发〔2021〕17号）

各省、自治区、直辖市及新疆生产建设兵团卫生健康委、发展改革委：
　　为贯彻落实党的十九大和十九届二中、三中、四中、五中全会精神，深入实施《[国务院办公厅关于促进3岁以下婴幼儿照护服务发展的指导意见](https://www.pkulaw.com/chl/434b24b8b5769383bdfb.html)》（国办发〔2019〕15号）和《[国务院办公厅关于促进养老托育服务健康发展的意见](https://www.pkulaw.com/chl/d1ad8fb179db9915bdfb.html)》（国办发〔2020〕52号），经全国评比达标表彰工作协调小组同意，国家卫生健康委、国家发展改革委决定开展全国婴幼儿照护服务示范城市（以下简称示范城市）创建活动。
　　一、总体要求
　　贯彻落实党中央、国务院决策部署，充分发挥政策和规划的引导作用，创新管理体制，健全服务机制，以满足人民群众对婴幼儿照护服务的需求为目标，以普惠服务为重点，建立完善促进婴幼儿照护服务发展的政策法规体系、标准规范体系和服务供给体系，调动社会力量的积极性，多种形式开展婴幼儿照护服务，形成一批可复制、可推广的典型经验，探索一批切实管用的政策举措。到2025年，婴幼儿照护服务的政策法规和标准规范体系基本健全，每千人口拥有3岁以下婴幼儿托位数达到4.5个，人民群众的婴幼儿照护服务需求得到进一步满足。

　　二、创建范围
　　示范城市创建以设区的市（地、州、盟）、直辖市的区（县）为单位开展，评选工作每2年为一个周期，每周期各省（区、市）推荐的示范城市不超过可参评单位总数的10%。

　　三、创建内容
　　（一）完善支持政策。将婴幼儿照护服务纳入国民经济和社会发展规划，制定“十四五”托育服务体系建设专项规划或实施方案，明确托育服务体系建设目标、建设任务、资金来源和运营方式等。制定“一老一小”整体解决方案，加强组织实施，方案含金量及实施的情况将作为考评的重要方面。综合运用规划、土地、住房、财政、投资、融资、人才等支持政策，大力发展成本可负担、方便可及的普惠性托育服务。在年度建设用地供应计划中保障托育用地需求，并安排在合理区位。支持将各类房屋和设施用于发展托育，鼓励适当放宽最长租赁期限。非独立场所按照相关安全规定标准改造建设托育点并通过验收的，无需变更土地和房屋性质。托育服务各项税费优惠政策全面、及时惠及市场主体，对吸纳符合条件劳动者的托育机构按规定给予社保补贴。加强婴幼儿发展与健康管理、婴幼儿保育等学科建设，培养相关专业人才。支持保险机构开发相关责任险及托育机构运营相关保险。
　　（二）扩大服务供给。全面落实产假政策，探索试行与婴幼儿照护服务配套衔接的育儿假、产休假。通过入户指导、亲子活动、家长课堂等方式，为婴幼儿家庭提供经常性的、普惠可及的育儿指导，提高家庭科学育儿能力。将婴幼儿照护纳入城乡社区服务范围，加强社区婴幼儿照护服务设施与社区卫生等设施的功能衔接，鼓励开展家庭互助式服务。新建住宅小区与配套婴幼儿照护服务设施同步规划、同步建设、同步验收、同步交付，在城市居住社区建设补短板和城镇老旧小区改造中统筹推进婴幼儿照护服务设施建设。发展集中管理运营的托育服务网络，建设一批承担指导功能的普惠性托育机构，支持产业园区、用人单位等在工作场所为职工提供福利性托育服务，婴幼儿入托率、千人口托位数高于全省平均水平。
　　（三）推动创新融合。深化医育有机结合，加强对托育机构卫生保健工作的业务指导和人员培训。充分利用互联网、大数据、物联网、人工智能等技术，研发应用婴幼儿照护服务信息管理系统，推进“互联网＋托育服务”，支持优质托育机构平台化发展。开发科学育儿公益课程、父母课堂等，提供互联网直播互动式家庭育儿服务。加强公共场所母婴设施的建设和改造，开辟绿色通道，为婴幼儿出行、哺乳等提供便利条件。培育托育服务、乳粉奶业、动画设计与制作等行业民族品牌。
　　（四）完善监管服务。落实政府在制度建设、行业规划、行政执法等方面的监管责任。强化部门协同，卫生健康行政部门牵头，相关部门按照各自职责，定期对托育机构开展监督检查。健全托育机构备案登记制度、信息公示制度和质量评估制度，落实托育机构的安全管理主体责任。发挥群团组织和行业组织的作用，加强宣传教育和社会监督，强化行业自律。依法实施守信联合激励和失信联合惩戒，构建以信用为基础的新型监管机制。托育机构服务质量和声誉口碑好，婴幼儿家庭满意程度高，无安全责任事故。

　　四、工作要求
　　（一）加强组织领导。各省（区、市）卫生健康委、发展改革委要把示范城市创建活动作为贯彻落实党中央、国务院决策部署以及促进婴幼儿照护服务高质量发展的重要抓手，主要领导亲自抓，分管领导具体抓，明确相关部门责任，统筹协调、上下联动，广泛深入地开展创建活动。
　　（二）制定创建方案。要按照本通知要求，结合本地实际，制定具体创建方案，明确目标任务，细化评估内容，建立激励机制，积极组织党委政府重视程度高、工作基础条件好、创新意识强、群众需求大的城市进行申报。
　　（三）认真组织实施。要加强创建活动全过程的调研指导，坚持好中选优、宁缺勿滥，严格评审标准和程序，确保创建活动的质量和生命力。认真总结示范城市的经验做法，鼓励因地制宜、创新发展，加强宣传与推介，营造良好社会氛围。
　　各省（区、市）卫生健康委、发展改革委要高度重视、认真组织好第一批示范城市评选工作。2021年9月30日前，将创建活动方案报送国家卫生健康委、国家发展改革委。2022年9月30日前，将推荐的示范城市申报材料报送国家卫生健康委、国家发展改革委；2022年年底前，国家卫生健康委、国家发展改革委将命名第一批全国婴幼儿照护服务示范城市。
　　联 系 人：国家卫生健康委人口家庭司　陈晨
　　联系方式：010-62030586
　　附件：全国婴幼儿照护服务示范城市创建活动管理办法

国家卫生健康委
　　国家发展改革委
　　2021年4月30日

　　附件

全国婴幼儿照护服务示范城市创建活动管理办法

　　第一条　根据《[国务院办公厅关于促进3岁以下婴幼儿照护服务发展的指导意见](https://www.pkulaw.com/chl/434b24b8b5769383bdfb.html)》（国办发〔2019〕15号），为规范全国婴幼儿照护服务示范城市创建活动，制定本办法。

　　第二条　全国婴幼儿照护服务示范城市是国家卫生健康委、国家发展改革委授予在促进婴幼儿照护服务发展中成效明显、群众满意，能够发挥示范引领、带动辐射作用的城市的荣誉称号。

　　第三条　国家卫生健康委负责创建活动的总体协调，会同国家发展改革委制定管理办法，组织公示和命名。省级卫生健康委、发展改革委组织本辖区创建活动，制订工作方案，细化评估内容，负责评审推荐。

　　第四条　全国婴幼儿照护服务示范城市创建活动坚持公开公正、好中选优的原则，按照城市主动申报、省级评审推荐、国家审核命名的程序进行。
　　（一）城市主动申报。有意愿的城市人民政府向省级卫生健康委、发展改革委提交申报报告。
　　（二）省级评审推荐。省级卫生健康委会同发展改革委对申请城市进行评审，确定候选名单，公示后上报国家卫生健康委、国家发展改革委。
　　（三）国家审核命名。国家卫生健康委会同国家发展改革委对申报材料进行审核，必要时进行实地考察。审核结果通过国家卫生健康委、国家发展改革委门户网站公示10个工作日，接受社会监督。通过审核公示的，国家卫生健康委、国家发展改革委予以命名并发文通报。
　　（四）动态管理。对于已命名示范城市，国家卫生健康委会同国家发展改革委适时组织开展复查，符合条件的继续保留命名。

　　第五条　已命名示范城市存在下列情形之一的，由国家卫生健康委、国家发展改革委撤销命名：
　　（一）发生重大安全责任事故的；
　　（二）措施不力，工作退步，存在弄虚作假等情形的；
　　（三）托育机构、婴幼儿家庭对政策环境和服务管理满意度不高的。

　　第六条　已命名的示范城市按照以下程序退出：
　　（一）出现第五条所列情形的，由省级卫生健康委、发展改革委调查核实，并将核实情况上报国家卫生健康委、国家发展改革委。
　　（二）国家卫生健康委、国家发展改革委研究决定撤销命名，并通过适当方式向社会公布。
　　（三）被撤销示范城市称号的，取消下一期参加评选资格。

　　第七条　省级卫生健康委、发展改革委每年向国家卫生健康委、国家发展改革委报送创建活动以及全国婴幼儿照护服务示范城市工作情况。

　　第八条　本办法由国家卫生健康委、国家发展改革委负责解释，自印发之日起施行。

© 北大法宝：（[www.pkulaw.com](https://www.pkulaw.com/)) 专业提供法律信息、法学知识和法律软件领域各类解决方案。北大法宝为您提供丰富的参考资料，正式引用法规条文时请与标准文本核对。
欢迎查看所有[产品和服务](https://www.pkulaw.com/helps/2.html)。法宝快讯：[如何快速找到您需要的检索结果？](https://www.pkulaw.com/helps/70.html)[法宝](https://www.pkulaw.com/helps/69.html)V6有何新特色？

扫描二维码阅读原文

**原文链接：**<https://www.pkulaw.com/chl/3c950081cd4ea1a4bdfb.html>
